# Liraglutide + PYY_3-36_ Combination Therapy Mimics Effects of Roux-en-Y Bypass on Early NAFLD Whilst Lacking-Behind in Metabolic Improvements

**DOI:** 10.3390/jcm11030753

**Published:** 2022-01-30

**Authors:** Valentin Metzner, Gloria Herzog, Tobias Heckel, Thorsten Bischler, Julia Hasinger, Christoph Otto, Martin Fassnacht, Andreas Geier, Florian Seyfried, Ulrich Dischinger

**Affiliations:** 1 Division of Endocrinology and Diabetes, Department of Internal Medicine, University Hospital Würzburg, 97080 Würzburg, Germany; metzner_v@ukw.de (V.M.); hasinger_j@ukw.de (J.H.); fassnacht_m@ukw.de (M.F.); 2 Institute of Pathology, University of Würzburg, 97080 Würzburg, Germany; gloria.herzog@uni-wuerzburg.de; 3 Core Unit Systems Medicine, University of Würzburg, 97080 Würzburg, Germany; heckel.t@web.de (T.H.); thorsten.bischler@uni-wuerzburg.de (T.B.); 4 Department of General, Visceral, Transplant, Vascular and Pediatric Surgery, University Hospital Würzburg, 97080 Würzburg, Germany; otto_c@ukw.de (C.O.); seyfried_f@ukw.de (F.S.); 5 Division of Hepatology, Department of Internal Medicine, University Hospital Würzburg, 97080 Würzburg, Germany; geier_a2@ukw.de

**Keywords:** liraglutide, GLP-1, peptide tyrosine tyrosine (PYY), peptide tyrosine tyrosine 3-36 (PYY_3-36_), RYGB, gastric bypass, obesity, NASH, NAFLD

## Abstract

Background: Treatment options for NAFLD are still limited. Bariatric surgery, such as Roux-en-Y gastric bypass (RYGB), has been shown to improve metabolic and histologic markers of NAFLD. Glucagon-like-peptide-1 (GLP-1) analogues lead to improvements in phase 2 clinical trials. We directly compared the effects of RYGB with a treatment using liraglutide and/or peptide tyrosine tyrosine 3-36 (PYY_3-36_) in a rat model for early NAFLD. Methods: Obese male Wistar rats (high-fat diet (HFD)-induced) were randomized into the following treatment groups: RYGB, sham-operation (sham), liraglutide (0.4 mg/kg/day), PYY_3-36_ (0.1 mg/kg/day), liraglutide+PYY_3-36_, and saline. After an observation period of 4 weeks, liver samples were histologically evaluated, ELISAs and RNA sequencing + RT-qPCRs were performed. Results: RYGB and liraglutide+PYY_3-36_ induced a similar body weight loss and, compared to sham/saline, marked histological improvements with significantly less steatosis. However, only RYGB induced significant metabolic improvements (e.g., adiponectin/leptin ratio 18.8 ± 11.8 vs. 2.4 ± 1.2 in liraglutide+PYY_3-36_- or 1.4 ± 0.9 in sham-treated rats). Furthermore, RNA sequencing revealed a high number of differentially regulated genes in RYGB treated animals only. Conclusions: The combination therapy of liraglutide+PYY_3-36_ partly mimics the positive effects of RYGB on weight reduction and on hepatic steatosis, while its effects on metabolic function lack behind RYGB.

## 1. Introduction

Today, it is assumed that about one-quarter of the world’s population suffers from non-alcoholic fatty liver disease (NAFLD) [1]. This is part of a continuing global trend driven by the obesity pandemic [2,3,4,5]. Among other mechanisms, central links between both conditions seem to lie in emerging adipose tissue dysfunction and dysregulation of liver-adipose tissue interaction in obesity [2,6,7]. NAFLD is associated with a high prevalence of other metabolic disorders (including metabolic syndrome, diabetes mellitus and cardiovascular disease) but may also progress to advanced forms of the disease itself, such as non-alcoholic steatohepatitis (NASH), liver cirrhosis and hepatocellular carcinoma, resulting in elevated mortality [2,8,9,10,11,12].

Compared to other therapy options, RYGB has not only been shown to be the most effective in reducing weight [13,14,15,16] while improving glycaemic control [13,17,18] but also to improve the histological markers (e.g., steatosis, inflammation) of NAFLD or NASH [13,19]. However, the mechanisms of action behind these effects remain unclear. Following RYGB, significant alterations in bile acid cycling, vagus signalling, gut microbiome are seen and increased postprandial secretion of anorectic enteroendocrine hormones, especially peptide tyrosine tyrosine (PYY_3-36_) and glucagon-like peptide-1 (GLP-1) can be detected [14,16,20]. However, as the indication for RYGB-treatment is limited to a small group of people, an effective non-invasive therapy for NAFLD and NASH is urgently needed.

To date, there is no approved drug for either NAFLD or NASH [21,22,23,24]. However, studies focusing on the effect of gut hormone mimetics upregulated after RYGB are promising: First, liraglutide, a GLP-1-analogue has shown significant effects in the resolution of NASH without progression of fibrosis while significantly reducing BMI in a clinical phase 2 study [25]. Similar data on NASH resolution have been obtained for semaglutide in a recent phase 2b trial [26]. Second, PYY_3-36_ has shown its lowering effect on food intake in rodents and humans in a variety of studies, likely also recruiting higher cortical systems and appearing to act on a behavioural level [20,27]. However, data on its effects on NAFLD/NASH are missing. While single-target agonists are much less effective than RYGB, multi-peptide approaches have delivered promising results, as tirzepatide did most recently [28]. Furthermore, the promising potential of a combination treatment with PYY and liraglutide has been demonstrated several times with the main focus on weight loss and change of eating behaviour [29,30,31], but not yet on its effects on NAFLD.

In the present study, we addressed this gap by investigating the effects of combination therapy (liraglutide+PYY_3-36_) versus RYGB on an early form of NAFLD in a controlled manner adding to the three important findings we made using these treatments [31]: First, RYGB- and liraglutide+PYY_3-36_ treatment induce a persistent and significant weight loss, while sham, saline and PYY_3-36_ have no weight lowering effect and liraglutide-monotherapy only achieves a short-term weight loss with subsequent weight-regain. Second, only animals treated with RYGB or liraglutide+PYY_3-36_ consume significantly fewer calories. Third, only these animals show a significantly changed preference from HFD to low-fat-diet (LFD).

To evaluate the effects of these treatments on liver health, we performed a histologic assessment, measurements and calculations for HOMA-index (insulin and glucose) and adiponectin/leptin-ratio; the former to assess insulin resistance and the latter as it is considered a good indicator of adipose tissue dysfunction [32,33]. Being central inflammatory factors in early human NAFLD/NASH, *Tnf*- and *Il1b*-mRNA levels were measured using RT-qPCR [2,34,35,36]. *Fgf21* mRNA was measured, as it is typically upregulated in these diseases and likely represents a link between inflammation and metabolic dysregulation [34]. To identify possible pathways mediating effects of the different treatments, we performed RNA sequencing of liver samples using a well-established rat obesity model.

## 2. Materials and Methods

### 2.1. Animals, Drugs, Surgeries

As described before [31], obesity was induced in adult male Wistar rats (Charles River Laboratories, *n* = 58, 9–10 weeks old, initial body weight 329.8 ± 2.2 g) with a high-fat diet (C1090-60 HF diet, 5228 kcal/kg; 60% calories from fat, 16% from protein and 24% from carbohydrate; Altromin, Lage, Germany) for about 6 weeks. Then, the animals were randomized into six pharmaceutical/surgical treatment groups: Treatment with liraglutide s.c. (0.4 mg/kg/day, Victoza, Novo Nordisk Pharma, Bagsværd, Denmark) and isotonic saline (Braun, Melsungen, Germany) via osmotic minipump (*n* = 5). Treatment with PYY_3-36_ (0.1 mg/kg/day, Hölzel diagnostika, Cologne, Germany) via osmotic minipump and saline s.c. (*n* = 5). Treatment with liraglutide s.c. and PYY_3-36_ via osmotic minipump (*n* = 11) and treatment with saline only (via osmotic minipump and s.c., *n* = 9). RYGB was performed in 15 animals, sham operation in 13. See [37] for the description of RYGB and sham operation. All groups were monitored over a period of 24 days. During this period, the animals had free choice of HF- and an LF diet (C1090-10 LF diet, 3514 kcal/kg; 10% calories from fat, 24% from protein, and 66% from carbohydrate; Altromin, Lage, Germany) ad-libitum.

The local regulatory authority approved all procedures on animals in this study (Regierung von Unterfranken, Würzburg, Germany, AZ: 55.2-2532-2-467). The laws and directives of Germany and the European Union were followed in all experiments. (TierSchG, TierSchVersV, Directive 2010/63/EU).

Due to practical reasons in the experimental design, 6 animals of the PYY_3-36_+Liraglutide- and 3 of the saline-arm underwent a prolonged feeding period and were therefore excluded from calculation of Homeostasis Model Assessment (HOMA) as well as from blood transaminase-, histological- and RT-qPCR assessment. One saline-treated animal had to be excluded from the analyses because of sample freezing error and one RYGB-operated rat had to be excluded because it developed an unclear abdominal mass (See Supplementary Figure S1 for the group design).

### 2.2. Dissections and Sample Collection

After the 24-day observational period, isofurane-narcotised animals were sacrificed by cardiac puncture after an overnight fast and blood was transferred into tubes containing EDTA and a DPP IV inhibitor (DPP4, Merck, Darmstadt, Germany); afterwards, this plasma was quickly separated by centrifugation at 5 krpm for 10 min at 4 °C and stored at −80 °C as described before [37]. Livers were removed, frozen using liquid nitrogen and stored at −80 °C.

### 2.3. Liver Histology

Histologic assessment of liver samples was performed by a pathologist who was blinded to the study aims to evaluate steatosis, inflammation and fibrosis, which mark important stages in the development of NAFLD and its progression to non-alcoholic-steatohepatitis [2,22]. Liver samples were fixed in 4% neutral formalin at 20 °C after defrosting. The tissue was routinely processed for paraffin embedding and cross-sectioned to obtain 3 μm-thick sections. Sections were de-paraffinized, rehydrated, and processed for routine haematoxylin/eosin (H&E) and histochemical staining. Sirius red staining (not shown) was performed with aqueous picrosirius red staining solution (500 aqueous picric acid solution 1,2% with 100 mg sirius red). Because of a considerable presence of freezing artefacts partly impairing the identification of cell borders, we decided to assess macrovesicular- and microvesicular steatosis in percentage without the evaluation of hepatocyte hypertrophy as proposed by Liang et al. in a rodent specific NAFLD scoring system [38]. Inflammatory activity was assessed as described there [38]. Fibrosis was graded according to the NASH Clinical Research Network criteria (NASH-CRN) [39].

### 2.4. Enzyme-Linked Immunosorbent Assay

Plasma levels of insulin (ERINS; Thermo Fisher Scientific, Santa Clara, CA, USA), leptin (EK-003-17; Phoenix Pharmaceuticals, Mannheim, Germany) and adiponectin (80570; Crystal Chem, Elk Grove Village, IL, USA) were measured using rat specific assays. Measurements of blood glucose, aspartate transaminase (ASAT) as well as alanine transaminase (ALAT) were performed in an external laboratory (Laboklin, Bad Kissingen, Germany; on Cobas702, Roche). *HOMA* was calculated for the individual rat as follows:

HOMA=insulin(fasting)(mU/L)∗glucose(fasting)(mmol/L)/22.5

### 2.5. Liver Gene Expression Analysis

RT-qPCR was used to assess changes in liver inflammation. Therefore, liver samples were macrodissected in 10 mg organ samples. Total RNA was extracted using the Maxwell^®^ RSC simplyRNA Tissue Kit (AS1340; Promega, Fitchburg, WI, USA) and the Maxwell^®^ RSC Instrument (AS4500; Promega). Prior to extraction, tissues were homogenized using QIAGEN Tissue Lyser II (85300; QIAGEN, Venlo, The Netherlands) and further purified using Proteinase K (PK) Solution (MC5005; Promega). RNA was tested for integrity and concentration using NanoDrop™ 2000 c spectrophotometer (Thermo Fisher Scientific): We yielded an average of 441 ng/µL nucleic acid per sample with a mean A260/280-ratio of 2.12, which we reversely transcripted into cDNA using the QuantiTect Reverse Transcription Kit (205311; QIAGEN) and Mastercycler Gradient Instrument (Eppendorf SE, Hamburg, Germany). cDNA was stored at −20 °C until used for RNA sequencing or RT-qPCR.

RT-qPCR was performed with selected targets (*Tnf*, ID: Rn99999017_m1, GenBankSeq.: NM_012675.3; *Il1b*, ID: Rn00580432_m1, GenBankSeq.: NM_031512.2; *Fgf21*, ID: Rn00590706_m1, GenBankSeq.: NM_130752.1) using hydrolysis probes (TaqMan^®^; Thermo Fisher Scientific) and a CFX96™ Real-Time PCR Detection System (Bio-Rad, Hercules, CA, United States) on duplicates. Then, the efficiency of probes and Cq of samples was calculated using LinRegPCR software (V. 2020.0). Duplicates with ∆Cq > 0.8 were repeated once and excluded if they failed this requirement again. In addition to *Actb* (ID: Rn00667869_m1, GenBankSeq.: NM_031144.3), *Ubc* (ID: Rn01499642_m1, GenBankSeq.: BC103477.1) was used as a reference gene to improve stability as internal control [40]. To allow the use of efficiency-correction and normalization to multiple reference genes, further analysis was performed with the software qBase+ version 3.2 (Biogazelle, Gent, Belgium) based on normalization methods published by Hellmanns et al. [41] to calculate normalized relative quantities (NRQs). As NRQs are usually log-normal distributed [42], we proceeded with the statistical analysis with log-transformed NRQ values using GraphPad Prism version 9.1.2 for Windows (GraphPad Software, La Jolla, CA, USA). We tested for variance, significance and normality using analysis of variance- (ANOVA)-, and D’Agostino–Pearsons omnibus K2-test, and Tukey’s multiple comparisons test for multiple comparison correction where appropriate.

### 2.6. RNA Sequencing

As described before [43], RNA quality was checked using RNA 6000 Nano kits on a 2100 Bioanalyzer (Agilent Technologies, Santa Clara, CA, USA). Afterwards, cDNA libraries were prepared from total RNA with oligo-dT capture beads for poly(A)-mRNA enrichment using the TruSeq Stranded mRNA Library Preparation Kit (Illumina, San Diego, CA, USA) according to manufacturer’s instructions. After 15 cycles of PCR amplification, barcoded cDNA libraries were quantified via Qubit 2.0 Fluorometer using dsDNA HS Assay Kits (Thermo Fisher Scientific). Quality and size distribution (average size: 290 bp) was checked with a 2100 Bioanalyzer using DNA 1000 kits (Agilent Technologies).

Sequencing of pooled libraries was performed at 23–38 million reads/sample in single-end mode with 75 nt read length on the NextSeq 500 platform (Illumina) with 1% PhiX control library using High Output sequencing Kits v2.5. Demultiplexed FASTQ files were generated with bcl2fastq2 v2.20.0.422 (Illumina).

Illumina reads were quality- and adapter-trimmed using Cutadapt version 2.5 with a cutoff Phred score of 20 in NextSeq mode, discarding reads without any remaining bases (command line parameters: --nextseq-trim=20 -m 1 -a AGATCGGAAGAGCACACGTCTGAACTCCAGTCAC). Resulting high-quality reads were subsequently aligned to the rat genome (GCF_000001895.5/Rnor_6.0 primary assembly and mitochondrion) using STAR v2.7.2b [44] with default parameters based on RefSeq annotation version 106 for GCF_000001895.5/Rnor_6.0. Read counts were assessed on exon level and summarized for each gene via featureCounts v1.6.4 from the Subread package [45]. In the process, multi-mapping and multi-overlapping reads were quantified strand-specific and reversely stranded with a fractional count for each alignment and overlapping feature (command line parameters: -s 2 -t exon -M -O --fraction). Raw read counts were used to identify differentially expressed genes using DESeq2 [46] version 1.24.0. Read counts were normalized by DESeq2 and fold-change shrinkage was applied by setting the parameter “betaPrior=TRUE”. Differential expression of genes was assumed at an adjusted *p*-value (*padj*) after Benjamini–Hochberg correction < 0.05 and |log2FoldChange| ≥ 1. Pathway analysis was conducted using the GSEA function of clusterProfiler [47] version 3.12.0 for gene set enrichment analysis based on the Kyoto Encyclopedia of Genes and Genomes (KEGG) pathways.

## 3. Results

### 3.1. Histological Assessment: All Treatments Significantly Improved Liver Steatosis Compared to Placebo

At the end of the observational period, animals in the placebo groups presented with marked steatosis (percentages of macrovesicular and microvesicular steatotic liver cells: 41.5 ± 15.4% in the sham-group and 54.8 ± 21.8% in the saline-group), while the other groups showed significantly less steatotic hepatocytes (3.1 ± 2.0% (RYGB), 20.5 ± 19.0% (PYY_3-36_), 6.5 ± 6.6% (liraglutide) and 18.8 ± 7.2% (liraglutide+PYY_3-36_)).

**Figure 1:**
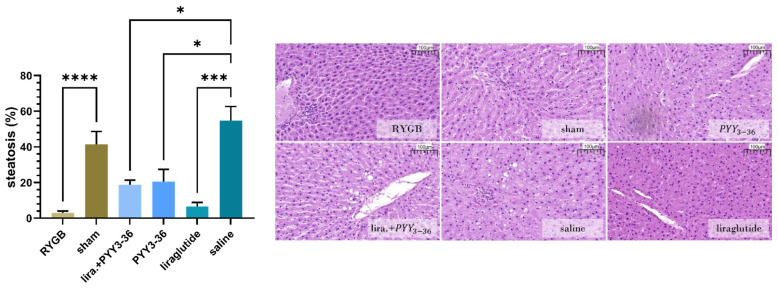
RYGB, PYY_3-36_ and/or liraglutide significantly improved liver steatosis. Steatotic load as percent (%) of liver cells affected by macrovesicular and microvesicular fat deposits of RYGB (*n* = 14), sham (*n* = 13), liraglutide+PYY_3-36_ (*n* = 5), PYY_3-36_ (*n* = 5), liraglutide (*n* = 5) and saline (*n* = 5). Levels of significance: * *p* ≤ 0.05, *** *p* ≤ 0.001 and **** *p* ≤ 0.0001. Data are presented as mean ± standard error of the mean. Scale inserted on histologic slides represent 100 μm; 20 × magnification.

As visualized in Figure 1 (effect of intervention: F(5, 41) = 13.09, *p* < 0.0001) reduction in steatosis was most prominent in the RYGB-group compared to sham (*p* < 0.0001), followed by liraglutide only (*p* < 0.001), liraglutide+PYY_3-36_ (*p* < 0.05) and PYY_3-36_ only (*p* < 0.05) compared to saline. No significant differences were detected between sham and saline or between RYGB, liraglutide+PYY_3-36_, PYY_3-36,_ and liraglutide. Considering only macrovesicular steatosis, it was not present in RYGB-animals and marginal in the other treatment groups (1.1 ± 0.8% (sham), 1.8 ± 2.3% (saline), 2.5 ± 3.9% (PYY_3-36_), 0.6 ± 0.7% (liraglutide) and 1.3 ± 0.3% (liraglutide+PYY_3-36_)).

No inflammatory foci or fibrosis were observed in any of the experimental groups.

### 3.2. RYGB Improved HOMA-IR- (Insulin Resistance) and Adiponectin/Leptin-Ratio

**Figure 2:**
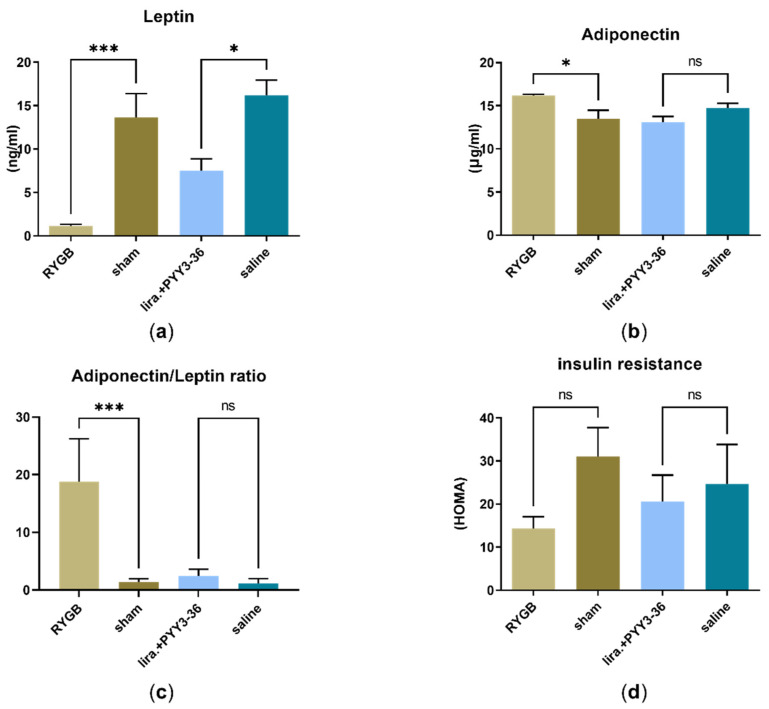
Metabolic improvements in RYGB vs. sham and liraglutide+PYY_3-36_ vs. saline: (**a**) Leptin (ng/mL) (RYGB: *n* = 8; sham: *n* = 10; liraglutide+PYY_3-36_: *n* = 11; saline: *n* = 7), (**b**) adiponectin (μg/mL) (RYGB: *n* = 10; sham: *n* = 7; liraglutide+PYY_3-36_: *n* = 6; saline: *n* = 4), (**c**) adipose tissue dysfunction (adiponectin/leptin ratio) (RYGB: *n* = 4; sham: *n* = 4; liraglutide+PYY_3-36_: *n* = 6; saline: *n* = 2) and (**d**) insulin resistance (HOMA) (RYGB: *n* = 4; sham: *n* = 7; liraglutide+PYY_3-36_: *n* = 5; saline: *n* = 4). Levels of significance: * *p* ≤ 0.05 and *** *p* ≤ 0.001. Data are presented as mean ± standard error of the mean.

Surgical (vs. sham) and combined medical (vs. saline) treatment had a significant impact on leptin levels (F(3, 32) = 11.56, *p* < 0.0001), as shown in Figure 2a. Leptin levels in RYGB treated animals were significantly lower than in sham (1.1 ± 0.5 ng/mL (RYGB) vs. 13.7 ± 6.2 ng/mL (sham), *p* < 0.001) and so were levels in the liraglutide+PYY_3-36_-group compared to the saline-group (7.5 ± 3.0 ng/mL (liraglutide+PYY_3-36_) vs. 16.2 ± 4.3 ng/mL (saline), *p* < 0.05). Second, adiponectin levels were significantly altered (F(3, 23)) = 6.15, *p* < 0.01) and significantly higher in RYGB treated animals (16.2 ± 0.3 μg/mL (RYGB) to 13.5 ± 2.4 μg/mL (sham), *p* < 0.05) and tendentially lower in the liraglutide+PYY_3-36_-group (13.1 ± 1.7 μg/mL (liraglutide+PYY_3-36_) to 14.7 ± 1.8 μg/mL (saline), *p* = 0.4) (see Figure 2b). Third, this resulted in significant changes in the adiponectin/leptin-ratio (F(3, 12) = 19.94, *p* < 0.0001) of the RYGB-group (18.8 ± 11.8 (RYGB) to 1.4 ± 0.9 (sham), *p* < 0.001) and a slight non-significant tendency towards a higher ratio in the liraglutide+PYY_3-36_-group (2.4 ± 1.2 (liraglutide+PYY_3-36_) to 1.2 ± 7.3 (saline), *p* = 0.9) (see Figure 2c). Fourth, regarding insulin-resistance- (HOMA-) index, indices were tendentially lower both in the RYGB group compared to sham (14.4 ± 8.6 (RYGB) to 31.0 ± 16.4 (sham); average insulin: 33.3 ± 17.1 µIU/mL (RYGB) and 55.5 ± 27.5 µIU/mL (sham); average glucose: 11.9 ± 1.6 mmol/L (RYGB) and 12.8 ± 2.0 mmol/L (sham)). Indices were also lower in the liraglutide+PYY_3-36_-group compared to saline (20.5 ± 17.1 (liraglutide+PYY_3-36_) to 24.7 ± 29.3 (saline); average insulin: 32.3 ± 29.8 µIU/mL (liraglutide+PYY_3-36_) and 40.2 ± 38.3 µIU/mL (saline); average glucose: 14.6 ± 4.6 mmol/L (liraglutide+PYY_3-36_) and 11.5 ± 1.7 mmol/L (saline)) but ANOVA revealed no significant differences (F(3, 16) = 1.10, *p* = 0.4), see Figure 2d.

### 3.3. No Significant Differences in Transaminase Levels (ALAT and ASAT)

ANOVA of serum ALAT- levels (24.7 ± 4.4 U/L (RYGB), 18.6 ± 4.1 U/L (sham), 21.1 ± 8.8 U/L (liraglutide+PYY_3-36_), 17.4 ± 3 U/L (liraglutide), 20.3 ± 7 (PYY_3-36_), 21.0 ± 6.4 (saline)) revealed no significant difference between the treatment groups (F(5, 35) = 1.59, *p* = 0.2). Furthermore, no significant difference was found (F(5, 36) = 1.06, *p* = 0.4) regarding serum levels of ASAT (34.9 ± 7.6 U/L (RYGB), 39.4 ± 6.9 U/L (sham), 50.5 ± 41.1 U/L (liraglutide+PYY_3-36_), 44.4 ± 18.1 U/L (liraglutide), 31.7 ± 14.0 (PYY_3-36_), 46.8 ± 36.8 (saline)).

### 3.4. No Inflammatory Activity Both in RT-qPCR- and Histological Assessment

**Figure 3:**
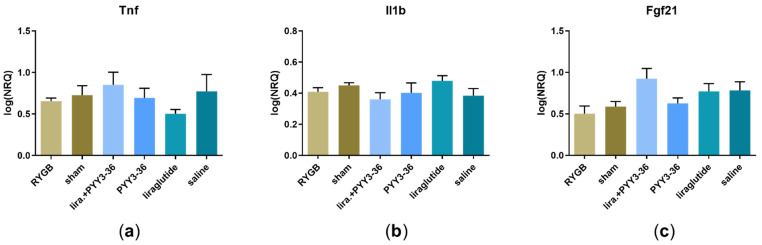
RT-qPCR assessment of the inflammatory markers in liver samples (**a**) *Tnf* (RYGB: *n* = 8; sham: *n* = 4; liraglutide+PYY_3-36_: *n* = 3; PYY_3-36_: *n* = 3; liraglutide: *n* = 3; saline: *n* = 2), (**b**) *Il1b* (RYGB: *n* = 8; sham: *n* = 8; liraglutide+PYY_3-36_: *n* = 4; PYY_3-36_: *n* = 4; liraglutide: *n* = 5; saline: *n* = 3) and (**c**) *Fgf21* (RYGB: *n* = 8; sham: *n* = 9; liraglutide+PYY_3-36_: *n* = 5; PYY_3-36_: *n* = 5; liraglutide: *n* = 4; saline: *n* = 3) as log-transformed normalized relative quantities (NRQs). Data are presented as mean ± standard error of the mean.

RT-qPCR-analysis revealed no significant difference in expression for *Tnf* (F(5, 17) = 1.32, *p* = 0.3) and *Il1b* (F(5, 26) = 1.44, *p* = 0.2), as shown in Figure 3a,b, but it did for *Fgf21* (F(5, 27) = 3.01, *p* < 0.05). However, post-hoc analyses showed no significant effects on *Fgf21* levels of treatment- vs. placebo- and between treatment-groups (Figure 3c).

These results are consistent with the histological examination in which we found no inflammatory foci in any of the samples.

### 3.5. Only RYGB Impacts Global Liver mRNA Expression

**Figure 4:**
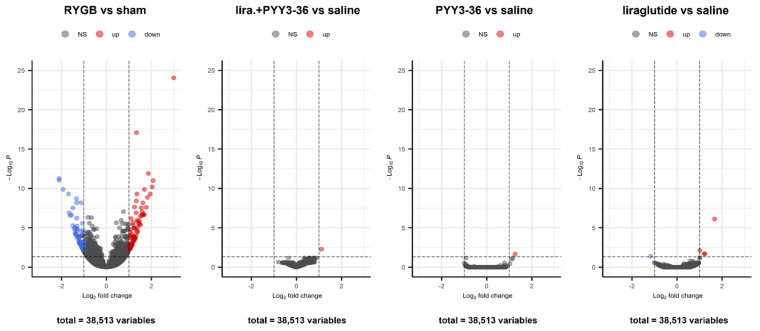
Volcano plots of differentially expressed genes in liver samples of RYGB vs. sham-treated animals, liraglutide + PYY_3-36_ vs. saline treated animals, PYY_3-36_ vs. saline treated animals and liraglutide vs. saline treated animals (RYGB: *n* = 14; sham: *n* = 12; liraglutide+PYY3-36: *n* = 11; PYY3-36: *n* = 5; liraglutide: *n* = 5; saline: *n* = 8).

**Figure 5:**
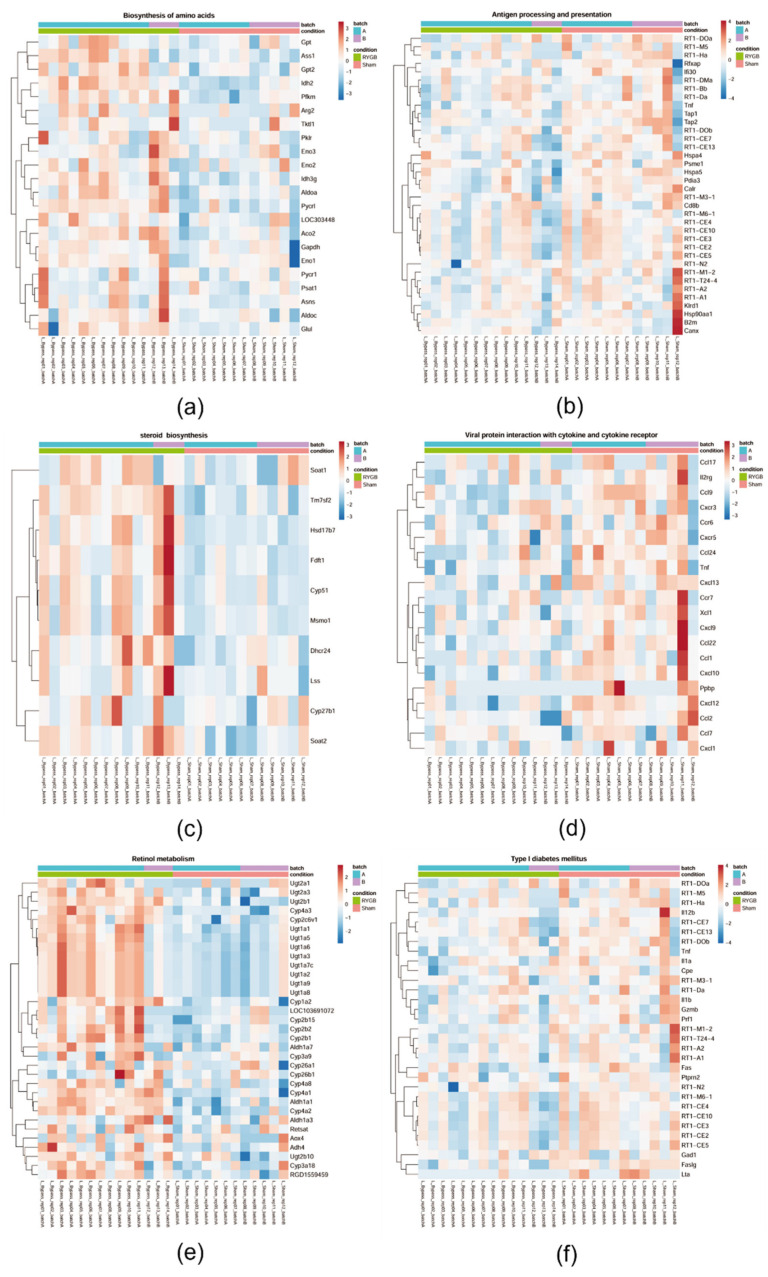
Heatmaps depicting gene expression levels in liver samples of RYGB vs. sham-treated animals for the GSEA-based core enrichment of selected significantly up- (**left**) or down-regulated (**right**) KEGG pathways (*p.adj* ≤ 0.05). Expression values represent row-wise z-scores of VST-transformed read counts with higher expressed genes in red and lower expressed genes in blue. GESA, gene set enrichment analysis. (**a**) biosynthesis of amino acids, (**b**) antigen processing and presentation, (**c**) steroid biosynthesis, (**d**) viral protein interaction with cytokine and cytokine receptor, (**e**) retinol metabolism and (**f**) type I diabetes mellitus.

While no relevant differences in liver mRNA expression were found in PYY_3-36_, liraglutide+PYY_3-36_ and liraglutide only treated animals (compared with saline controls), a high number of genes were differentially regulated (up 76, down 67) in RYGB treated animals compared with sham controls (Figure 4). Gene set enrichment based pathway analysis revealed an upregulation of the following selected pathways (with *p.adj* ≤ 0.05): ribosome, retinol metabolism, aminoacyl-tRNA biosynthesis, biosynthesis of amino acids, and steroid biosynthesis (Figure 5a,c,e). Proinflammatory chemokines (viral protein interaction with cytokine and cytokine receptor), antigen presentation pathways and pro-apoptotic pathways (Type I diabetes mellitus) were downregulated (Figure 5b,d,f).

See Table S1 for the pathways analyzed and their statistics.

## 4. Discussion

### 4.1. Treatment Effects Regarding Steatosis Are Comparable between Liraglutide+PYY_3-36_ and RYGB

Using a controlled study design, we have demonstrated the promising potential of the combined regime (liraglutide+PYY_3-36_) as an alternative to RYGB in the treatment of obesity, as shown in a previous publication [31] and as summarized in the introduction. As obesity is strongly associated with NAFLD [2,3,4,5] and its reduction with subsequent improvement of liver inflammation and fibrosis [48], we performed a histological evaluation of this condition: In our study, hepatocellular steatosis was significantly reduced in the RYGB- and all medical treatment groups; most prominently in the RYGB group. As RYGB as well as liraglutide+PYY_3-36_ treated animals show a significantly reduced overall food intake and a reduced high-fat preference [31], composition of consumed diet and total energy intake undoubtedly is a relevant cause for the improved liver health in these treatment groups.

Although leading to a more pronounced body weight loss [31], the impact of the combination therapy of liraglutide+PYY_3-36_ on liver steatosis did not differ from liraglutide monotherapy. However, another point worthy of note is that PYY_3-36_-monotherapy treated animals did not show alterations of caloric intake and body weight compared to respective control [31] but presented with mitigated NAFLD as well—a novel finding, which we will pursue in the future using a high-affinity Y2-receptor-agonist.

### 4.2. RYGB Controls Metabolic Dysregulation

In addition to the fact that NAFLD or its progression to NASH must be reversed or halted if increased mortality from liver-related events such as hepatic decompensation or hepatocellular carcinoma is to be prevented [49,50,51], control of metabolic dysregulation must also be sought. First, adipose tissue dysfunction has to be reverted as it not only orchestrates these changes in liver and beyond but also may be central in disease development [2,6,7]. Second, insulin resistance has to be overcome as it is the strongest predictor of progressive metabolic disease [52].

Our animals fall into this critical treatment period, showing pronounced steatosis without fibrotic changes in the histological assessment. Inflammatory activity in histology and RT-qPCR and therefore NASH was not yet present. After four weeks of treatment, control of adipose tissue dysfunction in the RYGB-treated animals was seen, as the adiponectin/leptin ratio significantly increased (Figure 2c), which is consistent with recent publications [32,33,53]. In contrast, the animals treated conservatively with combination therapy only showed a significant (and somewhat less pronounced) decrease in their leptin—without a significant change in their adiponectin levels—resulting in a slight non-significant increase in this ratio (Figure 2a–c). Insulin resistance appears to be improved by RYGB (Figure 2d), as this procedure usually does [13,17,18]. Although, the HOMA index was formally not significantly reduced due to high standard deviation. However, this effect appears to be apparently smaller in the liraglutide+PYY_3-36_ group compared to saline. Although proven by histology, NAFLD was not accompanied by altered transaminase levels (ASAT and ALAT). This supports the mostly negative recommendation of international NAFLD guidelines regarding screening by these parameters [54]. The performance of this non-invasive test seems limited [55] even in the case of advanced fibrosis in NAFLD patients [56], and up to 80% of human NAFLD patients may have normal ALAT levels [57]. Interestingly, only RYGB led to a high amount of differentially regulated genes compared to sham, as measured via RNA sequencing of liver samples (Figure 4). This probably underlines the high potential of RYGB as a multi-target intervention. The downregulated pathway “viral protein interaction with cytokine and cytokine receptor“ shows mainly down-regulated chemokines in RYGB vs. sham (Figure 5d). This underlines possible positive effects of RYGB on liver health, as population-based studies indicate that elevation of proinflammatory chemokines may be associated with NAFLD [58,59]. The “Type I diabetes mellitus” pathway shows mainly downregulated MHC class I genes and apoptosis genes such as *Fas*, *Faslg*, *Tnf* (not confirmed in RT-qPCR) (Figure 5f). This could indicate that RYGB reduces the effect of the immune system as a driver of NAFLD [60,61]. Retinol metabolism is known to be disturbed in NAFLD [62,63]. Although, it is unclear if this is a cause or consequence of this disease. To the best of our knowledge, for the first time, we can link these changes to RYGB specifically, as there was no relevant number of up- or downregulated genes in animals under an (in terms of body weight loss) equally effective medical treatment (liraglutide+PYY_3-36_) (Figure 4). Further studies have to go more into detail regarding this finding. In contrast to previous works suggesting an influence of liraglutide on liver ER stress pathways [64], we found no differentially regulated genes in the liraglutide-treated animals. However, the effectivity of liraglutide is shown, as treated animals lost weight [31].

Additionally, as both treatments (liraglutide+PYY_3-36_ and RYGB) had shown comparable effects on weight reduction, food intake, preference [31] and hepatic steatosis (Figure 1), this might indicate that the effects in the RYGB group are the consequence of the known sudden but long-lasting change in physiology, whereas the effects of the liraglutide+PYY_3-36_ treatment reflect an ongoing gradually building drug effect.

### 4.3. Limitations and Strengths of the Present Study

The short follow-up period of 4 weeks is a limitation of the present study, eventually preventing a more precise statement about the different effects on metabolic parameters of combined drug versus surgical treatment. Furthermore, the HOMA index alone is inferior to functional glucose tolerance measurements. Using both methods would have allowed a more accurate statement regarding the insulin resistance of the animals. Moreover, the animal model of HFD-induced obesity in rats contains distinct advantages and disadvantages: on the one hand, rats on HFD develop marked obesity within a short period of time and show greater biochemical similarities with humans, especially compared to mice [65,66]. Furthermore, compared to diets with modified amino acids or genetic models of adiposity, HFD-models mimic the Western diet, which is seen as one of the most relevant drivers of the obesity pandemic [67,68,69]. On the other hand, animals under HFD usually do not show advanced fatty liver disease states such as with severe inflammation or marked fibrotic changes of the liver, as found in human alimentary NAFLD/NASH, even after longer feeding periods, although inflammatory aspects of progressive NAFLD can be detected [66]. Our experimental animals were obese, with a bland NAFLD and metabolic impairment indicative of an early stage of the disease. To further investigate the effects of the treatments used on more advanced forms of fatty liver disease, further research on more advanced NASH animal models is warranted; combined HF + high sugar diets over an extended time frame seem to induce more inflammatory and fibrotic changes [66]. For these reasons, the present study is less meaningful concerning the more advanced inflammatory form of fatty liver disease. Given the 3R-Principle, we kept the number of study groups in this exploratory pilot study at its minimum, balancing animal suffering and the significance of results as described [31]. This might negatively influence statistical power on some occasions.

### 4.4. Synopsis

The unabated obesity pandemic will dramatically increase the burden of associated diseases, especially NAFLD and NASH in the near future [2,4,5,67,70,71,72,73]. Time is pressing to implement effective socio-economic measures in terms of primary prevention—many of which could also help mitigate another problem synergistically interconnected with global malnutrition: the climate crisis, one part of the global “Syndemic” [74]. Therefore, while obesity rates continue to rise, effective treatment options are still rare and, similar to bariatric surgery, limited to a relatively small group of people. For this reason, the need for effective conservative treatment options for NAFLD is greater than ever, but to date, none are approved [23,75]. We present here the data from one of the first controlled animal studies on the attenuation of histological NAFLD and metabolic parameters by combined administration of PYY_3-36_ and liraglutide. While the effects of liraglutide+PYY_3-36_ on NAFLD are comparable to the effects of RYGB, liraglutide+PYY_3-36_ has no significant effect on metabolic markers, contrary to RYGB. Further studies are needed to clarify whether there exists a gradually building drug effect that is beyond the scope of this study with its 4-week follow-up-period.

## Data Availability

The data discussed in this publication have been deposited in NCBI’s Gene Expression Omnibus (Edgar et al., 2002) and are accessible through GEO Series accession number GSE192425 (https://www.ncbi.nlm.nih.gov/geo/query/acc.cgi?acc=GSE192425) (last accessed on 28 December 2021). A token (for reviewers) can be obtained from the corresponding author.

## Supplementary Materials

The following supporting information can be downloaded at: https://www.mdpi.com/article/10.3390/jcm11030753/s1. Table S1: Results of KEGG pathway mapping on the basis of the comparison of liver mRNA results in RYGB vs. sham-treated animals. Figure S1: Schematic representation of the study groups, interventions and exclusion causes.
