# Supplementary material for: Liraglutide + PYY3-36 Combination Therapy Mimics Effects of Roux-en-Y Bypass on Early NAFLD Whilst Lacking-Behind in Metabolic Improvements
Source: J Clin Med. 2022 Jan 30;11(3):753. doi: 10.3390/jcm11030753 (PMC8836549; doi:10.3390/jcm11030753)
Supplement: Supplementary file 1 [file jcm-11-00753-s001.zip › jcm-1555396-Figure S1.pdf]

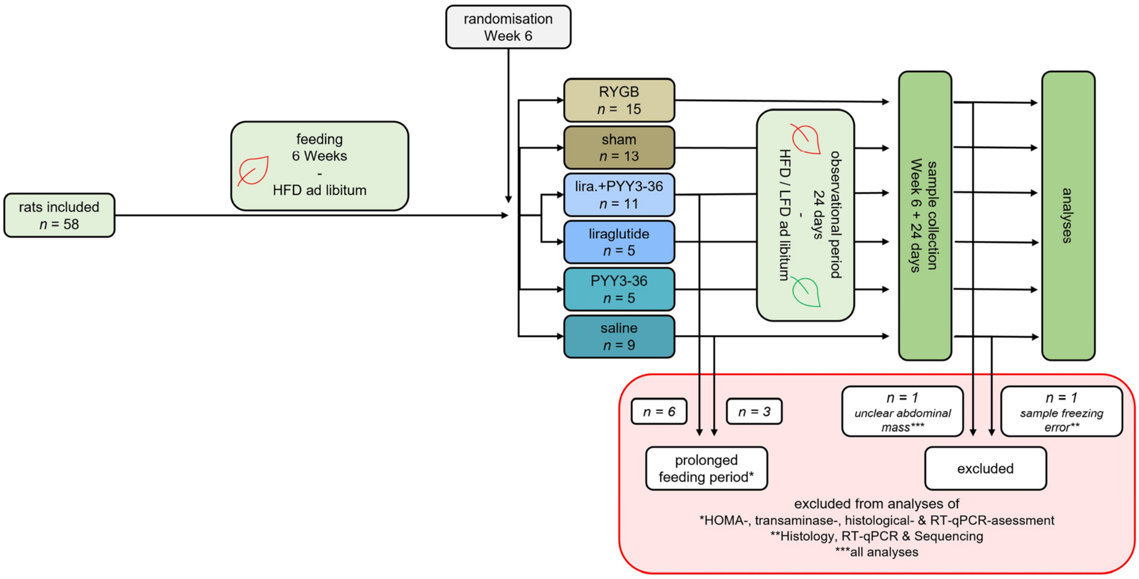

Supplementary Figure S1| Schematic representation of the study groups, interventions and exclusion causes
